# Hepatitis E Virus Infection in a Hospital from Southern Romania—New Data About a Threat to Public Health

**DOI:** 10.3390/microorganisms13102290

**Published:** 2025-10-01

**Authors:** Cristina Popescu, Alexandra Cireșă, Gabriel Adrian Popescu, Carmen Cristina Vasile, Leontina Mirela Bănică, Dragoș Florea

**Affiliations:** 1 Faculty of Medicine, “Carol Davila” University of Medicine and Pharmacy, 020021 Bucharest, Romania; 2 “Prof. Dr. Matei Bals” National Institute of Infectious Diseases, 021105 Bucharest, Romania

**Keywords:** acute hepatitis, hepatitis E virus in Romania, zoonotic transmission

## Abstract

This study analyses all cases of acute hepatitis E diagnosed in a southern Romanian hospital from 2019 to 2023. Patients with positive anti-HEV IgM antibodies were included in three groups: group A1—96 patients with probable HEV infection and ALT levels over 2.5-fold the upper limit of normal (ULN); group A2—44 patients with probable HEV infection and ALT levels under 2.5-fold ULN; group B—43 patients with probable HEV coinfection with another hepatotropic virus. Between 2019 and 2023, 642 patients were diagnosed with acute viral hepatitis. Positive anti-HEV IgM antibodies were detected in 183 (28.5%) cases, HEV being the second most common cause of acute viral hepatitis. Patients from group A were older than those from group B (47.26 ± 15.13 years vs. 35.95 ± 14.83 years, *p* < 0.01). Patients from group A were less likely to present clinical features compared to those from group B: digestive symptoms (73.8% vs. 97.2%, *p* < 0.01), jaundice (38.9% vs. 88.4%, *p* < 0.01), hepatomegaly (64.1% vs. 88.6%, *p* = 0.02). Patients from group A, compared to patients from group B, had lower levels of ALT (18.2 ± 29.9 ULN vs. 83.7 ± 56.2 ULN, *p* < 0.01) and total bilirubin (3.08 ± 5.2 mg/dL vs. 7.82 ± 5.25 mg/dL, *p* < 0.01) at admission. Patients from group A had higher levels of anti-HEV IgM antibodies (4.3 ± 3.22 vs. 2.55 ± 1.34, *p* < 0.01) than those from group B. Nearly all patients had no history of travel; therefore, autochthonous origin of HEV is involved in a large majority of cases. Currently, hepatitis E virus is not an uncommon aetiology of acute hepatitis in Romania, more often in adults and elderly patients. The epidemiological and clinical features of HEV infections plead for a zoonotic transmission in most cases. The significant number of cases of hepatitis E diagnosed in a single centre in Bucharest justifies the need to include early testing for HEV in patients with acute hepatitis.

## 1. Introduction

The number of acute viral hepatitis cases reported in Romania has substantially decreased over the last two decades [1]. This is probably the result of hepatitis B vaccination and the use of highly active direct-acting antivirals for the treatment of hepatitis C, sustained by national health programmes. However, other causes of acute hepatitis, such as hepatitis E, were not distinctly monitored until recently. The Romanian National Centre for Communicable Diseases Surveillance and Control published a Guidance for the surveillance of Hepatitis E in March 2024 [2].

Infections caused by the Hepatitis E Virus (HEV), officially named *Paslahepevirus balayani*, an RNA virus belonging to the *Paslahepevirus* genus [3], were rarely reported in Romania until 2023 [4,5,6]; this may be due to the low number of actual cases of hepatitis E, or to underdiagnosis of hepatitis E, both because of the paucity of symptoms, and also since testing for HEV in patients with acute hepatitis has been implemented by a limited number of hospital laboratories in Romania. The taxonomy of HEV was recently reviewed by the International Committee on Taxonomy of Viruses and the *Paslahepevirus* genus was allocated to the *Orthohepevirinae* subfamily, *Hepeviridae* family, *Hepelivirales* order, *Alsuviricetes* class [3]. Before 2022, hepatitis E cases were reported in the national surveillance system in the category of other acute viral hepatitis; consequently, no national data specific to HEV infections were published until 2023. Starting from 2022, hepatitis E was separately included in the list of infectious diseases with mandatory reporting, and all healthcare providers in Romania are required to report possible or confirmed cases of Hepatitis E.

An analysis by the World Health Organisation (WHO) indicated an increase in the global incidence of HEV infections from 2015 to 2022, with an estimated 20 million cases of HEV infections each year, and 3.3 million symptomatic cases [7]. Also concerning was the tenfold increase in HEV infections in the EU/EEA between 2006 and 2015, with a peak of 5500 cases in 2015 [8]. Most importantly, more than 95% of cases reported in Europe were autochthonous, with only a few cases associated with travel in endemic areas [9], in contrast to the previous period, when HEV infection was correlated with travelling in endemic areas. Currently, HEV is the most frequent aetiology for non-A, non-B, non-C acute hepatitis and the most common cause of acute hepatitis in the EU/EEA [10].

Moreover, the consumption of undercooked meat from infected pigs, and to a lesser extent, wild boars, is the main transmission route for HEV in European countries. The highest seroprevalence was found for two zoonotic types of HEV: mainly genotype 3 and, to a much lesser extent, genotype 4 [11]. A secondary but possible route of transmission of HEV in Europe is the transfusion of contaminated blood products [12].

Before 2010, a significant difference in the seroprevalence of anti-HEV antibodies existed between developing countries (with an estimated rate between 30% and 80%) and developed countries (with a rate between 1% and 20%); however, when the diagnostic assays were improved to detect also anti-HEV genotype 3 antibodies, the seroprevalence seems to be higher also in developed countries, reaching 40–42% in the USA and the UK and 52% in southwestern France [13].

Some seroprevalence studies on HEV infection have also been performed in Romania. In 2008, Săvuță et al. reported that the seroprevalence of anti-HEV IgG antibodies was 5.9% in a general population of one county from the North-Eastern region of Romania (Iași County) [14]. Another study conducted in 2010 in a different county of the same Region (Botoșani County) by Anița et al. reported a 12% seroprevalence rate of anti-HEV IgG antibodies in patients chronically infected with hepatitis B or C viruses [15]. The prevalence of anti-HEV IgG antibodies in other studies conducted in Romania varied between 12% and 28%, depending on the population subgroups tested and according to the population age [16,17]. A more recent study from North-Eastern Romania reported a seroprevalence of anti-HEV antibodies of 12.2% in 2022 [18]. The main risk factors for HEV infection identified by these studies published in Romania were the consumption of water from unsafe sources and improperly cooked meat [18].

Our study aims to describe the clinical and epidemiological characteristics of patients admitted for HEV infections at the largest hospital for infectious diseases in Romania, as well as the evolutive features that can differentiate these infections from other acute viral hepatitis. We consider this analysis particularly relevant because the number of patients with acute or chronic viral hepatitis assessed yearly in this hospital exceeds a couple of thousands. The ultimate goal of this study is to highlight the existence of HEV infections in Romania, so that physicians consider this aetiologic hypothesis more frequently than it is currently assumed, and that more laboratories develop the capability to confirm HEV infection.

## 2. Materials and Methods

A retrospective analysis of patients with positive results for anti-HEV IgM antibodies was conducted between January 2019 and December 2023 at the National Institute for Infectious Diseases “Prof. Dr. Matei Balș” in Bucharest, Romania.

### 2.1. Characteristics of the Study Groups

Each patient with a positive anti-HEV IgM antibody test in the previously mentioned period was included only once in the analysis, eliminating duplicate results. A total of 2180 anti-HEV IgM antibody tests were performed in the previously mentioned period, from which 276 tests were positive or equivocal (a positivity rate of 12.6%), with 239 positive tests and 37 equivocal tests. The 239 positive tests belonged to 205 patients (some patients were repeatedly tested). A number of 22 patients with a positive anti-HEV IgM antibody test, tested in our laboratory but admitted to other hospitals, were excluded from the analysis, since no clinical or laboratory data were available. The remaining 183 patients with positive anti-HEV IgM antibodies were included in the study.

For each patient, we used a standardised data sheet which contains demographic, clinical and laboratory data. These data were retrospectively collected using the patient’s clinical record from the hospital database. Acute hepatitis E with significant cytolysis was defined by biochemical and serological criteria: an ALT level at least 2.5 times higher than the upper limit of normal (ULN) and the presence of anti-HEV IgM antibodies. An “acute HEV infection without significant cytolysis” was defined as ALT levels below 2.5-fold ULN and positive anti-HEV IgM antibodies. We arbitrarily chose the threshold of 2.5-fold the ULN as the differentiation between acute hepatitis E and acute infection without significant cytolysis, because other studies of acute HEV infection also used this breakpoint [19,20].

The severity of acute hepatitis E was assessed using the prothrombin index (PI), and we defined severe acute hepatitis E by a PI lower than 50% or an international normalised ratio (INR) higher than 1.5 [21].

**Figure 1:**
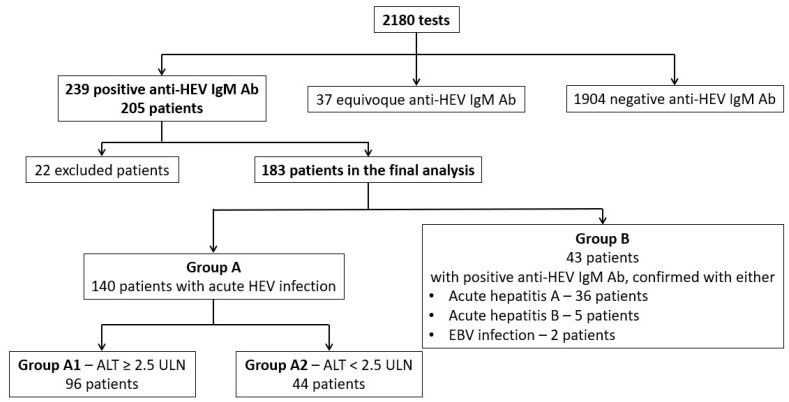
The groups of patients included in the study for the clinical analysis.

We divided the 183 patients into two groups (Figure 1):

- Group A—140 patients with probable acute HEV infection (positive anti-HEV IgM antibodies) and no positive tests for acute infections with any other hepatotropic virus;
- Group B—43 patients with probable acute HEV infection, but also with positive tests for other acute hepatitis viruses: anti-hepatitis A IgM antibodies (36 patients), anti-hepatitis B core IgM antibodies (5 patients), and positive IgM anti-viral capsid antigen of Epstein–Barr virus (EBV) (2 patients). We supposed that anti-HEV IgM antibodies could be false positive for some of these patients.

For an analysis of clinical manifestations, including the ALT level relevance for disease severity, we divided group A into two subgroups according to the value of ALT:

- Group A1 (significant cytolysis) with ALT higher than 2.5-fold ULN (96 patients);
- Group A2 (without significant cytolysis) with ALT lower than 2.5-fold ULN (44 patients).

### 2.2. Serological and Molecular Analysis

Anti-HEV IgM antibodies were detected by an enzyme-linked immunosorbent assay (DiaPro, Milan, Italy). Test results are interpreted as a ratio of the sample optical density and cut-off value (OD/CO). If OD/CO was below 1, the test was considered negative; if OD/CO was between 1 and 1.2, the test was considered equivocal, while if OD/CO was over 1.2, the test was considered positive. The patients with equivocal results needed to be retested after 1–2 weeks [22]. The detection of HEV-RNA by real-time RT-PCR was conducted using a commercial kit, Hepatitis E virus Genesig (Primer Design, Manchester, UK) which can detect less than 100 copies of target template.

### 2.3. Statistical Analysis

Data were collected from the hospital electronic database. We used SPSS Statistics 26 software for data analysis and Microsoft Office 2019 software for data collection and graphical figures. We used an independent *t*-test and a one-way ANOVA test for continuous variables, a Pearson Chi-Square test, Fisher’s Exact test and a Z-score for proportions for categorical variables. We considered the results to be statistically significant at a *p*-value < 0.05. Two-tailed tests measured the statistical significance.

## 3. Results

### 3.1. Epidemiological Characteristics of the Study Population

There were 642 patients diagnosed with acute viral hepatitis A-E between 2019 and 2023, out of which 183 were diagnosed with hepatis E. The yearly distribution of these acute viral hepatitis cases is displayed in Table 1. Hepatitis E was found in 28.5% cases of acute viral hepatitis between 2019 and 2023, the second rate after hepatitis A. The low number of acute hepatitis cases in 2020 and 2021 is most likely a result of the COVID-19 dedicated hospital status.

**Figure 2:**
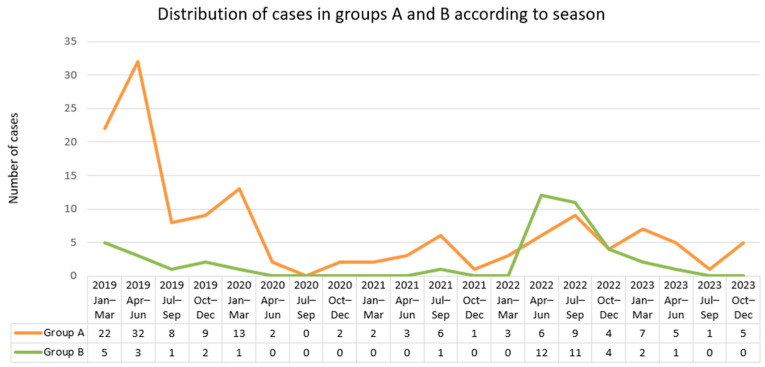
Quarterly distribution of cases in patients with probable acute HEV infection without (group A) or with (group B) markers for other hepatitis viruses.

The quarterly distribution of the 183 cases is illustrated in Figure 2. There were no statistically significant differences between the number of cases related to the season in which they were diagnosed for neither of the analysed groups: Group A (*p* = 0.58); Group A1 (*p* = 0.62); Group A2 (*p* = 0.57); Group B (*p* = 0.82); the whole lot (*p* = 0.62).

The demographic characteristics of patients are presented in Table 2.

Group A included significantly more women than group B (*p* < 0.01). The study groups included patients aged between 3 and 80 years old. Group A included older individuals compared to group B, with a mean age of 47.26 ± 15.13 years and 35.95 ± 14.83 years, respectively, *p* < 0.01. The patients with acute HEV infection and significant cytolysis (group A1) were older than patients with no significant cytolysis (group A2), with a mean age of 49.28 ± 15.13 years and 42.84 ± 14.34 years, respectively, *p* < 0.01. The study groups included 131 (73.2%) patients from an urban environment and 48 (26.8%) patients from a rural environment. Four patients were only travelling in Romania from other countries. There was no difference regarding patients’ environment between groups A and B (*p* = 0.33). Most of the patients, 113 (63.1%), were from the metropolitan area of Bucharest, while 66 (36.9%) were from other regions of the country. There was no difference regarding the region from which the patients came between groups A and B (*p* = 0.47). There were no significant differences between group A1 and group A2 in terms of sex, patient environment, or region of residence.

The study groups included ten patients who had a recent history of travelling to other countries: Greece (two patients), Ukraine (two patients), India (two patients), Denmark, Bulgaria, France, and Egypt (one patient each).

### 3.2. Clinical Characteristics

There were no patients in the study groups with a history of blood or blood-derived product transfusion in the last six months. A total of 43 patients had a history of immunosuppressive conditions: five patients with oncologic diseases (in group A), three patients with onco-haematological diseases (in group A), eleven patients with chronic hepatitis (eight in group A, three in group B), five patients with diabetes mellitus (four in group A, one in group B), sixteen patients with HIV infection (three in group A, 13 in group B), one pregnant woman (in group A), one patient with multiple sclerosis (in group A), and one patient with psoriasis (in group A).

The clinical features of patients included in the study are presented in Table 3.

Patients in group B were more likely to present with digestive symptoms (*p* < 0.01), jaundice (*p* < 0.01), and hepatomegaly (*p* = 0.02) compared to group A. Patients with significant cytolysis (group A1) were more likely to present jaundice at admission compared to other HEV-infected patients (group A2) (*p* = 0.01). There were no statistically significant differences between group A1 and group A2 regarding the presence of digestive symptoms, hepatomegaly, and fever.

There were three deaths registered in group A, and none in group B. Two patients were included in group A1, and one patient was included in group A2. All three patients had a history of chronic disease: two had chronic liver diseases, and one had diabetes mellitus.

### 3.3. Laboratory Characteristics

#### 3.3.1. Changes in Alanine Transaminase (ALT) Levels

The mean ALT levels at admission were 25.9 ULN (SD = 35.4 ULN) for group A1, 1.2 ULN (SD = 0.6 ULN) for group A2, 18.2 ULN (SD = 29.9 ULN) for the entire group A, and 83.7 ULN (SD = 56.2 ULN) for group B. The mean levels of ALT at admission were significantly higher in group B compared to group A [t(181) = 9.97, *p* < 0.01].

Group A1 included 96 patients. For 41 out of the 96 patients, we captured the moment of ALT normalisation. The mean length of time to ALT normalisation for group A1 was 47.37 days (SD = 46.5 days), ranging from 9 to 240 days. There were two patients who had high levels of ALT for more than 180 days, which can probably plead for a chronic HEV infection in those cases.

#### 3.3.2. Other Biochemistry Laboratory Markers

Table 4 displays the laboratory markers of patients included in the study.

The differences between groups A and B were not statistically significant, neither regarding the number of patients with a platelet count under 100.000 cells/mm^3^ (*p* = 0.68), nor regarding the number of patients with prothrombin index levels under 50% (*p* = 0.75). The mean bilirubin level was higher in patients from group B compared to patients from group A (*p* < 0.01). The differences between groups A1 and A2 were not statistically significant regarding the number of patients with a platelet count under 100.000 cells/mm^3^ or regarding the number of patients with prothrombin index levels under 50%. The mean bilirubin level was higher in patients from group A1 compared to those from group A2 (*p* < 0.01), the mean level of bilirubin being 3.95 mg/dL (SD = 5.98 mg/dL) and 1.18 mg/dL (SD = 1.75 mg/dL), respectively.

#### 3.3.3. Serological Features

From the 37 patients with equivocal anti-HEV IgM antibodies, 13 patients had also positive tests for acute infection with HAV, HBV or EBV, and 24 had no positive test for one of these viruses (15 patients with ALT higher than 2.5-fold ULN and nine patients with ALT lower than 2.5-fold ULN). The proportion of equivocal tests was 17.1% among patients with positive or equivocal anti-HEV IgM antibodies only and no markers for other hepatitis viruses (group A), and 30.2% for patients with a possible coinfection with HAV, HBV or EBV (group B); this difference is not statistically significant, z score = 1.86, *p* = 0.61.

We observed higher OD/CO levels of anti-HEV IgM antibodies in patients from group A compared to patients from group B [t(176) = 3.46, *p* < 0.01]. The mean OD/CO levels of anti-HEV IgM antibodies were: 4.94 (SD = 3.53) for group A1, 2.83 (SD = 1.62) for group A2, 4.3 (SD = 3.22) for group A, and 2.55 (SD = 1.34) for group B. Among patients from group A, higher OD/CO levels of anti-HEV IgM antibodies were registered in patients from group A1 compared to patients from group A2 [t(133) = 3.65, *p* < 0.01].

In a subset of cases, HEV-RNA was detected by real-time RT-PCR. The test was positive and confirmed the diagnosis in three cases from group A, and was undetectable in another 30 cases. The lack of detection of HEV-RNA does not rule out the diagnosis of acute hepatitis E, given the short duration of viremia in HEV infections.

## 4. Discussion

Hepatitis E virus was considered until recently as one of the leading causes of acute viral hepatitis in low- and middle-income countries, where prevention and control of this disease is difficult because of the limited access to clean drinking water, while in high-income countries, these cases occurred sporadically, mainly as a result of imported cases [7]. Over the last 20 years, HEV has become the leading cause of acute hepatitis worldwide due to the increased detection of HEV and the declining number of people newly infected with hepatitis A, B and C viruses [23,24]. The increased detection of HEV may be attributable to the increasing circulation of HEV, to the improvement of diagnostic tests and the rise in awareness among physicians [24].

During the first quarter of 2024, an increase in the number and severity of acute hepatitis E cases hospitalised at the National Institute for Infectious Diseases “Prof. Dr. Matei Balș” was observed. In this quarter, an unusually high number of cases of hepatitis E were diagnosed in our hospital: 17 cases, including one death. Eleven EU/EEA countries reported an increase in acute hepatitis E cases in January 2024. An update on the number of HEV cases detected in the EU/EEA was included in the Communicable Disease Threats Report published by the ECDC corresponding to week 6 of 2024, with the highest increase reported in Belgium, Czech Republic and Finland [25].

For this reason, we decided to retrospectively evaluate all cases of acute hepatitis E which were diagnosed between 2019 and 2023 in the largest hospital dedicated to infectious diseases in Romania, to detect a possible increase in the number of cases that might have been missed, especially because the reporting of HEV cases was not mandatory in Romania until 2024. Acute hepatitis E has seldom been diagnosed in Romania, possibly because of the low circulation of HEV, limited access to diagnostic tests, lack of awareness among clinicians, correlated to the absence of national surveillance data and national guidelines for diagnostic and treatment of hepatitis E. Consequently, only a few cases of hepatitis E in patients from Romania have been reported by the three studies published until 2024. Porea et al. identified 9 cases of acute HEV infection among 94 patients with acute hepatitis of unknown origin who were diagnosed over 10 months (May 2016–March 2017) at a hospital in North-Eastern Romania [4]. At the same hospital, Mihai et al. reported only 3 cases of acute HEV infection during 2018 and 2019. None of the patients had a history of international travel in the previous two months [5]. Finally, Istrate and Rădulescu described 48 cases of acute HEV infection admitted to a hospital in North-Western Romania over 32 months (January 2017–August 2019). Only one patient had a history of travelling outside Romania [6]. Although the origin of these cases might suggest that there is a localised spread of hepatitis E in the North-Eastern region of the country, our data mark the presence of hepatitis E in Bucharest and other areas from Southern Romania; this indicates at least a multiregional spread of hepatitis E in Romania; the implementation of an efficient surveillance system might be necessary to confirm this hypothesis and even more, a national spread of HEV.

Our data indicate that between 2019 and 2023, in the largest hospital for infectious diseases in Romania, hepatitis E was the second most frequent acute viral hepatitis, surpassed only by hepatitis A. We consider that the number of hepatitis E cases might have been even higher if testing for HEV had been performed more frequently in patients with acute hepatitis, which would have been in accordance with the European guidelines [26], even in the absence of epidemiological factors that are considered to be relevant (e.g., recent travelling in areas with endemic spread of HEV). The current study showed that all but a few cases had an autochthonous origin. This is similar to other EU/EEA countries over the last two decades and suggests a similar dominant route of transmission, i.e., zoonotic transmission [24]. Besides the autochthonous origin, this hypothesis is also supported by the evolution of spread, with sporadic cases (there were no descriptions of outbreaks of hepatitis E in our data, except for four cases with foreign origin), and the particular involvement of adults and elderly persons. Therefore, we believe that the results of our study support the necessity of raising awareness about HEV as a possible aetiology of autochthonous acute viral hepatitis, as well as the necessity of easily and continuously available serological and molecular tests for the efficient diagnosis of hepatitis E.

Globally, the route of transmission and risk factors for acute hepatitis E are genotype-dependent. HEV genotypes 1 and 2 are prevalent in developing countries and are linked to poor sanitation, while acute hepatitis E caused by genotypes 3 and 4 is mainly a zoonotic disease observed in developed countries [7]. The documented presence of HEV genotype 3 in the Romanian swine population [17], the absence of a travel history prior to disease onset and the prevalence of this genotype in the middle-aged and elderly population [27] suggest that the acute hepatitis E cases included in this study are likely related to HEV genotype 3. In our study, more than half (54.9%) of cases were identified in men, in accordance with Ijaz et al., who described a significant predominance of acute hepatitis E cases caused by genotype 3 in men (70%) [28].

The increased number of cases in the first quarter of the year could reflect consumer habits and customs in this region, such as an increased consumption of domestically prepared, undercooked or not thermally processed (e.g., dried, smoked) pork-derived products. The tradition of domestic slaughtering and butchering animals is an additional risk factor, as it increases occupational contact with pigs or wild boars in the winter season.

A higher number of acute hepatitis E cases were identified from January to March compared to the other months during the study period (2019–2023). However, no statistically significant differences were found regarding the quarterly distribution of cases. As well, a systematic review on the seasonality of hepatitis, published in 2015, did not identify a consistent seasonal pattern for acute hepatitis E [29]. The lack of statistical significance in our analysis may be attributed to the small sample size determined by the disruption in admissions caused by the COVID-19 pandemic. Indeed, during 2020, 2021 and the first six months of 2022, the hospital was dedicated almost exclusively to COVID-19 patients, and the number of acute hepatitis E cases admitted to the hospital was drastically reduced. Other possible explanations for the lack of seasonality may be related to a shift in the food habits of the general population from consuming traditional, domestically processed pork products to industrially processed products; also, the transmission of the virus through blood transfusion could drive year-round transmission of HEV [30], but this was not found in the current study.

The significantly younger population included in the group of patients with serological markers for HEV and other hepatitis viruses may be explained by the higher incidence of infectious mononucleosis and hepatitis A in younger age groups compared to hepatitis E, correlated to the possible false-positive results of anti-HEV IgM antibody assays in patients with acute EBV and/or HAV infections.

The greater proportion of patients presenting with clinical manifestations such as fever and jaundice in the group of patients with ALT values higher than 2.5-fold the ULN (group A1), compared to patients with lower ALT levels (group A2), may be due to the significantly older patients included in the first group. It is also possible that a higher percentage of patients from the first group had a pre-existing chronic liver disease, but data were not available to test this hypothesis.

Despite transfusion-related acute hepatitis E having been described in high-income countries, no patient included in our study presented a history of transfusion. This may be a result of the small sample size and the overall minor contribution of this transmission pathway to the disease burden.

Multiple studies have demonstrated discrepancies in the diagnostic sensitivity and specificity of various commercially available serological assays for anti-HEV antibodies [31,32,33], leading to low diagnostic accuracy.

We consider that an adequate diagnosis of acute hepatitis E must include either the detection of anti-HEV IgM antibodies and the detection of seroconversion or an increase in the titre of anti-HEV IgG antibodies. Alternatively, detection of anti-HEV IgM antibodies and detection of HEV-RNA in serum or stool could be used. To the best of our knowledge, IgG antibody serological assays have only been used in Romania for seroprevalence studies [4,5,6]. At the same time, the detection of HEV-RNA demands specialised laboratory facilities and is confined to a narrow window of no more than 4 weeks during the acute phase of the disease, in the majority of cases of acute hepatitis [34,35].

### Study Limitations

Our study was limited by the inability to measure anti-HEV IgG antibodies, which would have indicated prior seroconversion. Consequently, we were unable to identify false-positive results for anti-HEV IgM antibodies, especially in cases with serological markers for other hepatitis viruses (group B). This group included patients with apparent double infections; however, some of these patients may have had false-positive results for anti-HEV IgM antibodies. Another limitation of our study was the absence of HEV genotyping and the scarcity of testing for HEV-RNA, which could have clarified situations with false-positive serological assays (either for HEV or for EBV/HAV). Although indirect evidence exists, such as the endemic circulating genotype in the Romanian swine population, lack of a travel history and the main affected age groups, we cannot directly confirm that HEV genotype 3 was responsible for the cases included in our study.

## 5. Conclusions

The current study demonstrated that hepatitis E was the second most frequent acute viral hepatitis in the largest infectious disease hospital in Romania between 2019 and 2023, and that nearly all cases had an autochthonous origin. These data align with reports from other EU/EEA countries and with other studies about acute viral hepatitis in Romania, and suggest that autochthonous transmission of HEV may be more prevalent in Romania than previously considered. In addition, previous studies on circulating HEV genotypes in our country have reported sporadic cases, mainly distributed during the first months of the year, particularly among adults or elderly individuals. This indicates that most cases described in our study probably belong to HEV genotype 3.

Based on our results, we demonstrate the need for raising awareness among healthcare providers about HEV as a more common cause of acute hepatitis in Romania, and for availability of efficient tests for an accurate diagnosis of hepatitis E (i.e., serological assays with improved sensitivity for anti-HEV IgM antibodies, easier access to HEV-RNA tests, and serological assays for anti-HEV IgG antibodies in those cases where the diagnosis needs clarification). At the same time, most importantly, the promotion of safe food handling should be intensified as it represents the main preventive measure against the emergence of acute hepatitis E in the largely naïve Romanian population. Furthermore, the presence of such autochthonous cases of hepatitis E may justify the need for risk assessment of HEV transmission through blood transfusion and to include HEV in blood safety policies in our country. Moreover, depending on the evolution of the epidemiological context, in the case of a continuing increase in the incidence of HEV infection, active immunisation through vaccination against hepatitis E may be considered alongside non-specific preventive measures. Future studies are needed to identify at-risk population groups that would benefit from the vaccination.

## Figures and Tables

**Table 1:** The distribution of acute viral hepatitis cases according to the type of virus and year of diagnosis.

Aetiology	Year					Total
	2019	2020	2021	2022	2023	
HAV	75	13	7	142	55	292 (45.5%)
HBV	39	9	8	8	18	82 (12.8%)
HCV	14	2	0	4	1	21 (3.3%)
HDV	35	7	8	10	4	64 (9.9%)
HEV	82	18	13	49	21	183 (28.5%)
Total	245	49	36	213	99	642 (100%)

HAV = hepatitis A virus. HBV = hepatitis B virus. HCV = hepatitis C virus. HDV = hepatitis D virus. HEV = hepatitis E virus.

**Table 2:** Demographic features of patients included in groups A and B.

Demographic Feature	Group A	Group B	Group A vs. Group B
Sex (male) (N, %)	69 (49.3%)	32 (74.4%)	ꭓ^2^ = 8.4, *p* < 0.01
Age (years) (mean, SD)	47.26 (SD = 15.13)	35.95 (SD = 14.83)	t(181) = 4.3, *p* < 0.01
Urban environment (N, %)	102 (75%)	29 (67.4%)	ꭓ^2^ = 0.95, *p* = 0.33
Residence in the metropolitan area of Bucharest (N, %)	88 (64.7%)	25 (58.1%)	ꭓ^2^ = 0.6, *p* = 0.47

**Table 3:** Clinical features of patients included in groups A and B.

Clinical Feature	Group A (%)	Group B (%)	Group A vs. Group B
Digestive symptoms	69.6%	97.2%	ꭓ^2^ = 10.59, *p* < 0.01
Fever	64.8%	55.6%	ꭓ^2^ = 0.77, *p* = 0.38
Jaundice	31.8%	88.4%	ꭓ^2^ = 41.8, *p* < 0.01
Hepatomegaly	64.6%	88.6%	ꭓ^2^ = 6.16, *p* = 0.02

**Table 4:** Laboratory features of patients from groups A and B.

Laboratory Feature	Group A	Group B	Group A vs. Group B
PLT < 10^5^ cells/mm^3^ (N, %)	8 (5.8%)	1 (2.3%)	ꭓ^2^ = 0.85, *p* = 0.68
PI < 50% (N, %)	10 (8.1%)	4 (9.8%)	ꭓ^2^ = 0.11, *p* = 0.75
Total bilirubin (mg/dL) (mean, SD)	3.08 (SD = 5.2)	7.82 (SD = 5.25)	t(178) = 5.2, *p* < 0.01
ALP > ULN (N, %)	63 (49.2%)	37 (92.5%)	ꭓ^2^ = 23.69, *p* < 0.01
GGT > ULN (N, %)	106 (78.5%)	41 (95.3%)	ꭓ^2^ = 6.42, *p* = 0.01
Lipase > ULN (N, %)	15 (18.8%)	2 (6.9%)	ꭓ^2^ = 2.27, *p* = 0.23
Albumin < 3.5 g/dL (N, %)	8 (10.3%)	2 (7.7%)	ꭓ^2^ = 0.14, *p* = 1

PLT = platelet count. PI = prothrombin index. ALP = alkaline phosphatase. GGT = gamma-glutamyl transferase. ULN = upper limit of normal.

## Data Availability

The raw data supporting the conclusions of this article will be made available by the authors on request.

## Abbreviations

The following abbreviations are used in this manuscript:

ALP	Alkaline phosphatase
ALT	Alanine aminotransferase
CO	Cut-off
EBV	Epstein–Barr virus
ECDC	European Centre for Disease Prevention and Control
GGT	Gamma-glutamyl transferase
HAV	Hepatitis A virus
HBV	Hepatitis B virus
HCV	Hepatitis C virus
HDV	Hepatitis D virus
HEV	Hepatitis E virus
HIV	Human immunodeficiency virus
INR	International normalised ratio
OD	Optical density
PI	Prothrombin index
PLT	Platelet count
RNA	Ribonucleic acid
RT-PCR	Reverse transcription polymerase chain reaction
ULN	Upper limit of normal
WHO	World Health Organization
